# Access to dental services and use of toothpaste with optimal fluoride content in Peruvian children

**DOI:** 10.3389/fdmed.2024.1467501

**Published:** 2024-10-08

**Authors:** Natalie Hadad-Arrascue, María Claudia Garcés-Elías

**Affiliations:** Facultad de Estomatología, Universidad Peruana Cayetano Heredia, Lima, Peru

**Keywords:** dental care, toothpaste, health services accessibility, Peru, cross-sectional studies

## Abstract

**Background:**

Leading international health organizations advise using toothpaste that contains at least 1,000 parts per million of fluoride, emphasizing that this practice should begin with the eruption of the first primary tooth.

**Aim:**

To determine the association between access to dental services and the use of toothpaste with 1,000 ppm fluoride or more in Peruvian children under twelve years of age.

**Materials and methods:**

A cross-sectional analysis was conducted using data from the 2021 Demographic and Family Health Survey. The main variable studied was the use of toothpaste containing a fluoride concentration of 1,000 ppm or more. Additionally, the access to dental care, the time elapsed since the last dental visit, and the type of healthcare facility were analyzed. Multilevel regression was applied to examine the relationship between variables and the dependent variable, with Peru's 24 regions serving as the analytical level.

**Results:**

According to the bivariate analysis, the natural region, area of residence, place of residence, wealth index, and age were associated with the use of fluoride toothpaste with 1,000 ppm or more. In the multilevel analysis, the access to dental services was not associated with the use of fluoride toothpaste with 1,000 ppm or more (*p* = 0.454); similarly, the time elapsed since the last dental visit also showed no association (*p* = 0.676), as did the type of healthcare facility (*p* = 0.752, *p* = 0.896, *p* = 0.983).

**Conclusion:**

Accessing dental services, the time elapsed since the last dentist visit, and the location where that care was received were not associated with the use of toothpaste containing 1,000 ppm or more of fluoride among Peruvian children during the year 2021.

## Introduction

Several factors can prevent the development and progression of dental caries, with the practice of favorable behaviors like using fluoride toothpaste (FT) and regular toothbrushing standing out as key measures (1, 2). This combination is regarded as the primary preventive approach, independent of direct interventions by dentists, for maintaining good oral health. Consequently, many international organizations recommend using toothpaste with a minimum fluoride content of 1,000 parts per million (ppm). This practice should begin with the eruption of the first primary tooth, with its effectiveness maximized by applying it twice a day during toothbrushing (3, 4).

Since the World Health Organization includes FT in its model list of essential medicines, its quality and availability are crucial concerns for international public health (5). Among the critical factors associated with using FT are the financial capacity to purchase it regularly, the socio-cultural integration of its use into personal habits, product availability, and access to dental services (6, 7). In terms of the latter, timely dental care is essential for adopting a preventive approach and successfully developing beneficial oral health habits (8), such as using FT (9).

In Peru, the Clinical Practice Guidelines for the Prevention, Diagnosis, and Treatment of Dental Caries emphasizes the importance of a dental visit before the first year of life to identify a child's risk of caries and to educate parents or caregivers about oral health. This includes recommendations for home care, such as the use of FT. However, there are few nationally contextualized studies on the use of FT (7, 10) and its associated factors. Therefore, this research aimed to determine the association between access to dental services and the use of toothpaste with 1,000 ppm fluoride or more in Peruvian children under twelve years of age.

## Materials and methods

### Study design

A cross-sectional analysis was conducted using data from the 2021 Demographic and Family Health Survey (ENDES), managed by the National Institute of Statistics and Informatics of Peru (INEI). The ENDES survey is conducted annually by a rigorously trained team that performs in-home interviews and administers questionnaires to the selected population.

A two-stage, stratified, probabilistic, and independent sampling method was employed at the departmental level, covering both urban and rural areas. In 2021, the sample included 168,145 children under twelve years old. The final sample size, consisting of 24,057 children, was determined based on the respondents’ reports regarding the use of FT (measured in ppm). Incomplete records were excluded as part of the data management process. It is important to note that the information was provided by the individuals responsible for the children's healthcare (11). The ENDES survey sampling frame is based on the statistical and cartographic information from the 2017 National Population and Housing Censuses. The sampling units used are private households and clusters. In reference to two-stage sampling, the first stage involves selecting the primary sampling units (PSUs), which are the clusters, while the second stage involves selecting the secondary sampling units, which are the households. In two-stage household surveys, it is common to rotate clusters within the sample. Each year, a group of clusters is removed from the sample and another group is added, enabling continuous updating of the sampling process (11, 12).

### Variables

In this study, the dependent variable was defined as the use of toothpaste containing a fluoride concentration of 1,000 ppm or more. Question 839 from the ENDES health questionnaire states: “Please, could you show me the toothbrush and toothpaste that (NAME) uses to brush their teeth? Verify the fluoride concentration of the toothpaste and circle the corresponding code.” The possible responses pertain to the fluoride concentration in parts per million (ppm). The independent variables included access to dental care, the time elapsed since the last dental visit (measured in years), and the place of dental care. The type of healthcare facility providing the service was categorized as the Ministry of Health of Peru (MINSA), the Social Health Insurance of Peru (ESSALUD), the Armed Forces (FF.AA), the Police Forces (PNP), and the private sector. Additionally, covariates were delineated, including the natural region classified as Metropolitan Lima, the rest of the coast, the highlands, and the jungle.

Additionally, the area of residence was categorized into urban and rural settings, while the place of residence was classified as capital, small city, town, or countryside. Altitude was measured as either less than 2,500 meters above mean sea level (MAMSL) or 2,500 MAMSL and above. A wealth index was used to indicate each household's capacity to access and enjoy goods and services. This index applied a scoring system developed by the Demographic and Health Surveys Program of the United States, allowing for the classification of households into quintiles ranging from the least advantaged to the most advantaged (13, 14). Lastly, the variables included health insurance coverage, sex, and age.

### Procedures and statistics

Regarding the data acquisition process, the data were obtained from the official website of the National Institute of Statistics and Informatics (INEI) (http://iinei.inei.gob.pe/microdatos/), utilizing various modules and information sources. The data were then consolidated into a single matrix for analysis using STATA software, version 17. The statistical analysis involved calculating absolute and relative frequencies through descriptive methods. Chi-square tests were employed to investigate associations among the study variables. The “svy” command was used to ensure representative estimates aligned with the survey's design, incorporating sampling strata, primary units, and assigned weights.

Poisson logistic regression was used for multivariate analysis, determining crude (PR) and adjusted prevalence ratios (aPR) based on significant variables identified beforehand. Multilevel regression was applied to examine the relationship between variables and the dependent variable, with Peru's 24 regions serving as the analytical level. A variance component model (null model) was built using the dependent variable—use of toothpaste containing 1,000 ppm fluoride or more—without explanatory variables to assess overall variability attributed to regions, supporting subsequent analysis (*P* < .001).

Explanatory variables were then introduced to explore their associations with each independent variable and covariates. Four models were developed: unadjusted models 1, 2, and 3 for access to dental services, geographical characteristics, and sociodemographic factors, respectively, and adjusted model 4 for dental service access adjusted for covariates. It is important to note that the multilevel analysis performed accounted for the sampling method utilized in the study. A 95% confidence level and a significance level of *P* < 0.05 were applied uniformly across all analyses.

### Ethics

Regarding ethical considerations, the Institutional Ethics Committee of Universidad Peruana Cayetano Heredia granted approval on August 3, 2021. These datasets are publicly available and include coded records to ensure the anonymity of survey participants.

## Results

It must be stated that every frequency was analyzed independently. The use of toothpaste containing 1,000 ppm fluoride or more was reported by 81.46% (*n* = 4,507) of participants, while access to dental services was reported by 56.23% (*n* = 17,063). Only a minority, 12.64% (*n* = 1,985), indicated their last dental visit occurred less than two years ago, with the majority seeking care primarily from the Ministry of Health of Peru, accounting for 45.98% (*n* = 9,914) of visits (Table 1). In bivariate analysis, access to dental services, time since the last dental care, and the type of healthcare facility did not show significant associations with the use of toothpaste containing 1,000 ppm fluoride or more (*p* = 0.943, *p* = 0.800, and *p* = 0.166, respectively). Conversely, other sociodemographic factors such as natural region, area of residence, place of residence, wealth index, and age showed associations with the outcome (*p* < 0.001) (Table 2).

**Table 1:** Characteristics of children under 12 years old in Peru, 2021.

Variables	*n*	%
Use of fluoride toothpaste with 1,000 ppm or more		
Yes	4,507	81.46
No	19,550	18.54
Access to dental services		
Yes	17,063	56.23
No	14,960	43.77
Time since the last dental care		
Less than 2 years	1,985	12.64
2 years or more	14,987	87.36
Type of healthcare facility		
Ministry of health	9,914	45.98
Social security	1,790	12.07
Armed and police forces	36	0.55
Private sector	5,309	41.40
Natural region		
Metropolitan Lima	2,002	30.03
Rest of coastal regions	4,931	28.32
Highlands	5,397	25.46
Jungle	4,367	16.20
Area of residence		
Urban	11,370	77.43
Rural	5,327	22.57
Place of residence		
Capital	2,002	30.03
Small city	4,846	21.46
Town	4,522	25.94
Countryside	5,327	22.57
Altitude		
Less than 2,500 MAMSL	12,183	78.96
2,500 meters or more above mean sea level	4,514	21.04
Wealth index		
Very poor	4,457	20.47
Poor	4,209	23.18
Middle	3,153	21.91
Rich	2,390	18.99
Very rich	1,687	15.45
Health insurance coverage		
Yes	26,719	78.31
No	5,304	21.69
Sex		
Man	16,217	70.00
Woman	15,806	30.00
Age		
0–5 years old	16,832	50.42
6–11 years old	15,191	49.58

*n*: absolute frequency; %: relative frequency.

**Table 2:** Use of fluoride toothpaste with 1,000 ppm or more according to access to dental services and other characteristics in Peruvian children under 12 years old, 2021.

Variables	Use of fluoride toothpaste with 1,000 ppm or more				
	Yes		No		*p* *
	*n*	%	*n*	%	
Access to dental services					
Yes	2,430	81.43	10,875	18.57	0.943
No	2,077	81.51	8,675	18.49	
Time since the last dental care					
Less than 2 years	321	80.76	1,224	19.24	0.800
2 years or more	2,100	81.45	9,605	18.55	
Type of healthcare facility					
Ministry of health	1,290	83.07	6,249	16.93	0.166
Social security	314	76.34	1,131	23.66	
Armed and police forces	6	72.35	23	27.65	
Private sector	820	81.26	3,464	18.74	
Natural region					
Metropolitan Lima	375	78.24	1,028	21.76	<0.001
Rest of coastal regions	986	80.67	2,773	19.33	
Highlands	905	82.23	2,806	17.77	
Jungle	610	87.18	2,793	12.82	
Area of residence					
Urban	2,165	80.28	6,320	19.72	<0.001
Rural	711	85.69	3,080	14.31	
Place of residence					
Capital	375	78.24	1,028	21.76	<0.001
Small city	923	81.07	2,718	18.93	
Town	867	81.79	2,574	18.21	
Countryside	711	85.69	3,080	14.31	
Altitude					
Less than 2,500 MAMSL	2,123	81.36	7,063	18.64	0.687
2,500 meters or more above mean sea level	753	81.88	2,337	18.12	
Wealth index					
Very poor	510	88.00	2,638	12.00	<0.001
Poor	748	83.17	2,477	16.83	
Middle	638	78.76	1,760	21.24	
Rich	503	80.34	1,263	19.66	
Very rich	385	72.25	848	27.75	
Health insurance coverage					
Yes	3,724	81.73	16,460	18.27	0.408
No	783	80.44	3,090	19.56	
Sex					
Man	2,445	81.57	9,627	18.43	0.780
Woman	2,062	81.19	9,923	18.81	
Age					
0–5 years old	3,277	72.48	8,863	27.52	<0.001
6–11 years old	1,230	90.04	10,687	9.96	

*n*: absolute frequency%: relative frequency *p*: statistical significance.

* Chi-square test.

According to the multilevel regression analysis, access to dental services was not associated with the use of toothpaste with 1,000 ppm or more (*p* = 0.454); similarly, the time elapsed since the last dental visit also showed no association (*p* = 0.676), nor did the type of healthcare facility (*p* = 0.752, *p* = 0.896, *p* = 0.983) (Table 3).

**Table 3:** Multilevel analysis between the use of fluoride toothpaste with 1,000 ppm or more and access to dental services.

Variables	Use of fluoride toothpaste with 1,000 ppm or more								
	Null model	Unadjusted model				Adjusted model 4			
		Coefficient	PR	95% CI	*p*	Coefficient	aPRa	95% CI	*p*
Model 1: access to dental services									
Access to dental services									
No		Ref.				Ref.			
Yes		0.02	1.02	0.98–1.06	0.433	−0.02	0.98	0.94–1.03	0.454
Time since the last dental care									
Less than 2 years		Ref.				Ref.			
2 years or more		<0.01	1.00	0.92–1.09	0.971	0.02	1.02	0.94–1.11	0.676
Type of healthcare facility									
Ministry of health		Ref.				Ref.			
Social security (EsSalud)		−0.07	0.94	0.85–1.03	0.193	−0.02	0.98	0.88–1.09	0.752
FF.AA./PNP		−0.19	0.83	0.43–1.59	0.567	−0.05	0.95	0.47–1.93	0.896
Private sector		−0.01	0.99	0.93–1.06	0.744	<−0.01	1.00	0.93–1.08	0.983
Variance		0.12				0.10			
Intra-class correlation (ICC%)		0.01				0.86			
*p*		0.035				<0.001			
Model 2: Geographic characteristics									
Natural region									
Metropolitan Lima		Ref.							
Rest of coastal regions		<0.01	1.00	0.92–1.09	0.983		–	–	–
Highlands		0.03	1.03	0.95–1.12	0.439		–	–	–
Jungle		0.11	1.12	1.04–1.20	0.002		–	–	–
Area of residence									
Urban		Ref.							
Rural		0.08	1.09	1.04–1.14	<0.001		–	–	–
Place of residence									
Capital		Ref.							
Small city		0.01	1.01	0.91–1.12	0.834		–	–	–
Town		0.02	1.02	0.95–1.10	0.575		–	–	–
Countryside		0.10	1.11	1.03–1.19	0.004		–	–	–
Altitude									
Less than 2,500 MAMSL		Ref.							
2,500 meters or more above mean sea level		−0.03	0.97	0.92–1.03	0.363		–	–	–
Variance		0.10							
Intra-class correlation (ICC%)		0.86							
*p*		<0.001							
Model 3: Sociodemographic characteristics									
Wealth index									
Very poor		Ref.							
Poor		−0.09	0.92	0.87–0.97	0.002		–	–	–
Middle		−0.13	0.88	0.82–0.93	<0.001		–	–	–
Rich		−0.16	0.85	0.80–0.91	<0.001		–	–	–
Very rich		−0.20	0.82	0.76–0.89	<0.001		–	–	–
Health insurance coverage									
Yes		Ref.							
No		−0.02	0.98	0.93–1.03	0.531		–	–	–
Sex									
Man		Ref.							
Woman		−0.02	0.98	0.94–1.03	0.435		–	–	–
Age									
0–5 years old		Ref.							
6–11 years old		0.22	1.25	1.19–1.31	<0.001		–	–	–
Variance	0.18	1.28							
Intra-class correlation (ICC%)	0.02	0.23							
*p*	<0.001	<0.001							

PR, prevalence ratio; aRP, adjusted prevalence ratio. 95% CI: 95% confidence interval *p*: statistical significance.

^a^ Adjusted by natural region, area of residence, place of residence, wealth index and age.

## Discussion

This research reveals that as of 2021, the use of toothpaste with a fluoride content of 1,000 ppm or more among Peruvian children is not associated with their use of dental services, the time since their last dental care, or the provider of this care. These findings provide valuable insights into the oral health practices of Peruvian children.

In contrast, in 2018, Hernández-Vásquez and Azañedo (1) analyzed the ENDES survey and found that more than a quarter of Peruvian children who had received dental care in the six months before the survey used toothpaste with an inadequate fluoride concentration (less than 1,000 ppm); moreover, these differences were statistically significant. However, those results were merely descriptive and did not include additional regression analyses to determine the strength of the association. Another study applied to Peruvian children, comparing the years 2019 and 2020, reported that the use of toothpaste with at least 1,000 ppm increased in the last year. The factors associated with this outcome were the natural region, area of residence, place of residence, wealth index, and age (2).

Two studies conducted in Asia examined oral hygiene habits and their associated factors. The first, a nationally representative study, revealed that Chinese adolescents who had previously received dental care and had a positive attitude towards regular dental check-ups were more likely to brush their teeth twice a day with fluoride toothpaste (15); however, it did not specify if the amount of fluoride in the toothpaste was adequate. Similarly, the second study, focused on Japanese university students, identified that dental services are the primary source of knowledge about oral hygiene and the implements they use (16). The findings of this research contrast with the results obtained in other studies, indicating that the factors influencing toothpaste use among Peruvian children are not linked to access to the healthcare system.

A survey conducted in 136 countries between December 2005 and March 2006 indicated that the economic ability to purchase toothpaste was one of the main barriers to its use (6). This situation persists more than 19 years later (17). In this regard, a report revealed a wide variability in the prices of fluoride toothpaste, notably high for the poorest 15% of the population in Sub-Saharan Africa, as well as in regions of South and Southeast Asia and the Pacific Islands. In these territories, the cost of an annual supply of fluoride toothpaste per person could represent a catastrophic health expense (18), which could lead to foregoing its use or purchasing lower-priced options that may not have an adequate formulation to meet preventive purposes. Research conducted in 2021 revealed that Peruvian children from higher socioeconomic levels were less likely to use fluoride toothpaste (more than 1,000 ppm) compared to those from families in extreme poverty.

Coinciding with this point, Quevedo and Carrizales (19), in their analysis of ENDES for 2019, 2020, and 2021, found that the socioeconomic level of children in Peru influenced the use of toothpaste with optimal fluoride content. Children from economically disadvantaged households showed greater use of this product than those from higher quintiles. An explanation for these findings could be that higher social strata have greater access to various options regarding oral hygiene products, allowing them to consider more factors when purchasing these products. A study classifies the fluoride content in toothpaste as the main factor in its choice, followed by previous experience and, finally, a dentist's recommendation (20).

According to the geographic characteristics that showed statistically significant differences in this study, Mlenga and Mumghamba indicate that there are inequalities in toothpaste use between rural and urban sectors (21), as demonstrated in Malawi, a country in Africa where more than three-quarters of the population live in rural areas. However, this publication does not specify whether the toothpaste contained adequate parts per million of fluoride. This study found that residing in rural areas was positively associated with using toothpaste with fluoride of 1,000 ppm or more, compared to urban areas. However, in Saudi Arabia, no statistically significant differences were identified between the two sectors (22).

In 2019, it was observed that Peruvian children aged 0–5 mainly used toothpaste with less than 1,000 ppm fluoride (7). This trend has persisted, as this research reports a similar situation in 2021. Consequently, age appears to be a characteristic indicating vulnerability in Peruvian children. Given the results of a study conducted in the capital city (10), this finding reinforces the need for the state and health authority to ensure compliance with the guidelines established in their clinical practice guidelines to prevent dental caries and the marketing of products in the country, which is also supported by their regulation on toothpaste manufacturing (23).

Among the limitations of this research is the use of a database extracted from nationwide surveys, meaning that the responses provided by the participating population could present certain inaccuracies due to self-reporting. Additionally, a slight loss of data occurred for certain variables, as not all respondents answered every question posed by the interviewers during the administration of the ENDES survey. Nevertheless, this remains Peru's primary source of monitoring oral health indicators. Additionally, the established study type only allows the results to identify levels of associated factors and not causal relationships.

## Conclusion

Accessing dental services, the time elapsed since the last dentist visit, and the location where that care was received were not associated with the use of toothpaste containing 1,000 ppm or more of fluoride among Peruvian children during the year 2021.

## Data Availability

Publicly available datasets were analyzed in this study. This data can be found here: https://proyectos.inei.gob.pe/microdatos/.
